# Navigating NHS commissioning for digital mental health: a perspective on learning through collaboration

**DOI:** 10.3389/frhs.2025.1707463

**Published:** 2025-10-31

**Authors:** Charlotte L. Hall, Kelly-Marie Prentice, Olivia Hastings, Camilla M. Babbage, Sophie S. Hall, Sarah J. Bolton, Janet Bouttell, Jonathan Gibbons, Julian Patel, Michael Watts, E. Bethan Davies, Madeleine J. Groom, Chris Hollis

**Affiliations:** ^1^ Academic Unit of Mental Health & Clinical Neurosciences, School of Medicine, Institute of Mental Health, University of Nottingham, Nottingham, United Kingdom; ^2^ NIHR MindTech HealthTech Research Centre, School of Medicine, Mental Health and Clinical Neurosciences, Institute of Mental Health, University of Nottingham, Nottingham, United Kingdom; ^3^ NIHR Nottingham Biomedical Research Centre, Institute of Mental Health, University of Nottingham, Nottingham, United Kingdom; ^4^ Nottingham Clinical Trials Unit, School of Medicine, University of Nottingham, Nottingham, United Kingdom; ^5^ Centre for Healthcare Equipment and Technology Adoption, Nottingham University Hospitals NHS Trust, Nottingham, United Kingdom; ^6^ IP and Commercialisation Office, Research and Innovation, Ingenuity Centre, University of Nottingham, Nottingham, United Kingdom; ^7^ Health Innovation East Midlands, University of Nottingham Innovation Park, Nottingham, United Kingdom; ^8^ Blum Health Ltd., Birkenhead, United Kingdom; ^9^ Nottinghamshire Healthcare NHS Foundation Trust, Nottingham, United Kingdom

**Keywords:** digital mental health interventions, NHS commissioning, children and young people, tic disorders, implementation barriers and enablers, innovation adoption

## Abstract

Digital mental health interventions (DMHIs) offer promising solutions to address unmet mental health needs among children and young people, yet how to get DMHIs commissioned into the NHS can seem mystifying for innovators**.** This perspective paper draws on insights from a collaborative commissioning event focused on the Online Remote Behavioural Intervention for Tics (ORBIT) intervention, a digital behavioural therapy for young people with tic disorders, to explore the barriers and enablers to commissioning DMHIs in England. Key challenges identified include unclear commissioning pathways, limited clinical expertise, integration hurdles, and short-term funding models. Enablers included clinical advocacy, robust research evidence, and alignment with national frameworks. These insights highlight the importance of early collaboration between academics, developers, and policymakers in the product development cycle seeking to bridge the gap between innovation and implementation in digital mental health care.

## 1 Introduction

Digital mental health interventions (DMHIs) hold significant promise for improving access to support for mental health support for children and young people. They are often positioned as the solution to the growing demand for timely, effective, evidence-based mental health care which is not currently being met (1). Indeed, DMHIs offer the opportunity to provide effective mental health support at scale (2), reducing geographical boundaries, specialist clinician time and the need for families to travel to attend face-to-face appointments (3).

Broadly, DMHIs are developed by two groups: industry, and/or academic/clinical teams. Industry developers, often small- and medium-sized enterprises (SMEs), typically excel in technical development and commercialisation. However, industry sector developers may lack experience with clinicians and access to, or engagement with, service users. Industry developers also rarely conduct rigorous evaluations like randomised controlled trials (RCTs) unless they are awarded government grants, particularly as the National Institute for Health and Care Excellence (NICE) Evidence Standards Framework for digital health technologies requires that evidence for effectiveness must be shown, but does not require that this evidence is based on an RCT (4). NICE is an independent UK public regulatory body that develops evidence-based guidance and evaluates the clinical and economic value of health technologies to inform healthcare decision-making in the NHS.

Academic or clinical teams, by contrast, tend to address unmet clinical needs and produce evidence-based interventions with models for care integration, but often lack the skills and infrastructure to bring products to market. As a result, DMHIs with limited evidence are sometimes adopted in practice, while well-researched interventions remain stuck in development and unavailable to patients (5, 6). This results in patients missing out on effective, evidence-based interventions, while less robust products gain traction due to developers' stronger skills in marketing and commercialisation. In England, government-funded initiatives like Health Innovation Networks (HINs) support both academic and industry innovators by offering guidance on procurement systems, aligning interventions with NHS priorities, and connecting innovators with commissioners.

Although England's National Health Service (NHS) care is free at the point of use, commissioning digital interventions requires navigating complex regulatory and procurement frameworks to ensure public funds are spent appropriately and equitably. Following recent reforms, Integrated Care Boards (ICBs) now lead local service planning and funding, guided by Department of Health and Social Care (DHSC) priorities. Clinical leads, commissioners, and service managers also play key roles in evaluating interventions. In our view, decisions should be based on clinical and cost-effectiveness (including budget impact), user acceptability and engagement (7), regulatory compliance, and alignment with NHS priorities (8). In 2024, the British government set out three strategic shifts in the ten-year plan, to move from (1) hospital to community; (2) illness to prevention; and (3) analogue to digital (8). Alongside this, they developed a Strategic Commissioning Framework to support ICBs to strengthen their capability to drive these three priorities. Digital interventions play a key role in facilitating the realisation of these shifts, providing accessible care in non-clinical settings. Despite this, the commissioning and integration of such digital tools into routine care pathways remains complex. Innovators (both academic-led and industry) developing interventions for young people frequently encounter unclear pathways to adoption, fragmented commissioning structures, and limited opportunities to engage with decision-makers, even with the support of HINs. This lack of clarity in commissioning process has been highlighted within the DMHIs community as a barrier to adoption (9).

To better understand the challenges and potential solutions to DMHIs, we hosted a one-day workshop targeted at NHS commissioners and key decision-makers - such as clinical leads and service managers. The event focused specifically on the ORBIT intervention - an online, remotely delivered behavioural therapy designed for young people with tic disorders. ORBIT offers a scalable and evidence-based digital solution to address gaps in access to specialist care for young people with tic disorders (10). An RCT demonstrated ORBIT's clinical and cost-effectiveness (11–13), and the intervention recently underwent an Early Value Assessment (EVA) by NICE, resulting in conditional recommendation (14).

The workshop, held on 26th March 2025 at the University of Nottingham (UK), brought together NHS commissioners (*n* = 8), clinical (*n* = 1), regulatory (*n* = 2), commercialisation (*n* = 2), academic (*n* = 4), software development (*n* = 1), and lived experience (*n* = 1) experts, facilitated by 3 team members. Commissioners and decision-makers were invited via local HINs and professional networks. The event aimed to explore challenges, enablers, and best practices in commissioning digital mental health interventions, with a focus on cost-effectiveness and NHS adoption. Morning presentations aimed to brief attendees on the condition and intervention and care pathways, including information about tic disorders and sharing lived experience, with informal discussion encouraged through breaks. Afternoon roundtables focused on identifying barriers and solutions to adopting DMHI for young people. Where needed, travel and accommodation costs were covered for all attendees, including the lived experience member. Data were not formally analysed, but notes were taken and used to guide the team perspective.

This perspective summarises insights gained from the event, which were recorded via notes taken from key facilitators, who are co-authors of this paper. The perspectives and opinions of commissioners are often underrepresented in the academic literature. By sharing of our findings, we aim to bridge the gap between innovation and implementation, and offer practical considerations for researchers, developers, and policymakers seeking to ensure that effective digital tools reach the populations they are intended to support.

## 2 Barriers

Through our discussions as part of the workshop, three key barriers emerged to commissioning DMHIs.

### 2.1 Lack of clear pathways and expertise

The absence of established clinical pathways for both DMHI and for many mental health and developmental conditions (e.g., tic disorders) presents significant systemic challenges. Without national guidance (e.g., NICE Clinical Guidance), local commissioners and providers face uncertainty on how to best assess and treat conditions, leading to inconsistent service provision and reluctance to innovate. This is compounded by challenges integrating public health expertise with commissioning decisions - expertise that could provide critical insights into prevalence, population-level demand, and the value of prevention and early intervention. In the case of tic disorders - where services are rarely commissioned locally - this ambiguity contributes to a perceived risk of “opening the floodgates”, where introducing new services might overwhelm systems. While this risk is sometimes valid, it is not universally realised, particularly in the context of low-intensity digital interventions but could perversely lead to service providers receiving fines if a surge in demand significantly increases their waiting lists.

The lack of clinical expertise in the disorder in many geographical areas was also noted as a barrier. Where a diagnosis is required for a young person to be eligible to access the digital tool in healthcare services, healthcare professionals can lack the training or expertise to conduct the full assessments needed to provide that diagnosis. This issue is especially pronounced in areas like tic disorders, where comprehensive diagnostic evaluations rarely occur and highlights the issue of clinical gatekeeping which may delay or obstruct access to early, scalable digital interventions, undermining their potential to alleviate pressure on overstretched services. An additional barrier in such cases is the absence of existing service pathways, meaning that funding a new intervention often requires creating a new budget line without clear opportunities to offset costs elsewhere. For tic disorders, young people often find themselves referred between several services and “jumping through hoops” for months or even years before tangible support is offered, because the pathway is not clear.

### 2.2 Implementation and integration

Commissioners may hesitate to adopt DMHIs that require complex IT integration, workforce training, or service redesign - especially given funding pressures and unclear commissioning roles. Information governance requirements vary widely across NHS Trusts, with each having its own criteria for cybersecurity, data privacy, and impact assessments. This leads to duplicated work for innovators, inconsistent expectations, and delays. Although this process has been somewhat standardised by the NHS Digital Technology Assessment Criteria (DTAC), a standard that we and our partners are closely aligning, it has not removed all variability. DMHIs must also integrate with existing electronic patient record systems (where available) to ensure clinical activity is properly documented. This integration is often challenging, and providers must be equipped to support NHS sites. Initiatives like the Innovator Passport aim to streamline these processes and reduce variation across Trusts (15).

### 2.3 Locally-decided commissioning and lack of dedicated funding

Even if an intervention is considered clinically important and well evidenced, commissioners may not have allocated budgets to spend. Many ICBs are under financial pressure and are stretching relatively small budgets across a wide range of conditions and populations. Furthermore, planning is complex and variable with each ICB having its own local priorities, budget constraints and timescales in which budgets need spending. Even with support from HINs it can be difficult to scale across regions, as what gets funded in one area may not be accepted in another. Such regional disparities in funding can also exacerbate the “postcode lottery” of inequitable service provision across regions. Furthermore, funding may sometimes only be available on a short-term basis (e.g., one financial year). Like most interventions, DMHIs require sustained financial support (16), including for on-going licencing, integration with existing systems, ensuring compatibility with updated software, and evaluation. Commissioners may be unable or unwilling to commit to funding contracts beyond a short time frame, which in turn may make it difficult to realise the benefits of the intervention, particularly if they are not immediate or unlikely to be realised within the current financial year. Even if benefits are realised, it still does not guarantee that any budget would be available in the next year. As well as hampering the ability to demonstrate cost-effectiveness, this short-term funding approach may result in patients having to stop a DMHI that they are benefitting from.

## 3 Enablers

Our workshop was designed to focus on solutions, rather than barriers, in order to provide positive action points that can be taken forward. Subsequently, several key enablers were identified.

### 3.1 Clinical buy-in

Commissioners noted the importance of *clinical champions* to advocate for the adoption of a DMHI. Commissioners stated that when clinicians show strong support for the need to use an innovation to solve a provision problem, they listen. However, it was also acknowledged that since the COVID-19 pandemic, clinician endorsement alone is no longer sufficient if the case is not supported by cost-effectiveness evidence.

Commissioners noted that to get a “foot in the door”, innovators may wish to explore individual funding requests, which is a formal request made to the local ICB to fund a treatment, service, or intervention that is not routinely available through standard NHS commissioning pathways. These funding requests are reviewed by an expert panel, including clinicians, and competitor quotes can be gained by the committee to ascertain value for money and best fit. These requests are typically made by clinicians and can provide a test-case to evidence to the ICB the utility of providing the DMHI to their patient group which may help facilitate more sustainable financial commitment, though are only approved on a case-by-case basis.

### 3.2 Research and real-world evidence

The importance of research-based evidence - including RCTs demonstrating clinical and cost-effectiveness - was noted as important to supporting commissioning decisions. However, it is worth acknowledging that many DMHIs currently commissioned by the NHS have been adopted without RCT evidence. This suggests that while RCTs are valued, other factors such as compelling value propositions, strategic alignment, and effective stakeholder engagement may play a more influential role in real-world commissioning decisions. Importantly, commissioners noted that they considered the “whole service” impact when exploring benefits of interventions. Examples of what counted as meaningful clinical and service impact focussed on a broad range of outcomes, including symptom and service use reduction, as well as improved presenteeism in school or work for parents. Although the ORBIT intervention is positioned in secondary care, commissioners noted that demonstrating a reduction in use of primary and emergency care services was also important. They highlighted that interventions supporting the parent alongside the young person, and those that support patients awaiting a diagnosis are particularly important.

Alongside research evidence, commissioners noted the importance of case studies where NHS Trusts had implemented the intervention and demonstrated improvements to patient outcomes and their local budgets. In our workshop, our patient and public involvement (PPI) member shared their lived experience of their child's tic disorder and their struggle to get support. Commissioners commented how powerful this voice was and the importance of including this when developing a business case or sales pitch.

### 3.3 Clinical targets and frameworks

The value of receiving NICE guidance or recommendation was highlighted as a key enabler for the adoption of a DMHI. However, approval from NICE alone was not sufficient to guarantee uptake. In addition to NICE endorsement, other frameworks and standards were also cited as influential. For instance, Integrated Care Boards (ICBs) are required to meet the Mental Health Investment Standard annually, which mandates that their investment in mental health services must grow at a rate that matches or exceeds the growth of their overall NHS funding allocation (17). Another important framework mentioned was the i-THRIVE Framework, an implementation model designed to transform the way mental health services are delivered to young people in the UK (18). Digital therapies, such as ORBIT, align with the i-THRIVE Framework by delivering timely, needs-led support directly to children and families in their own communities through accessible digital therapy. Additionally, the importance of aligning with the government's “Three Shifts” was highlighted. ICBs are required to ensure their commissioning strategies reflect these shifts, and they will be evaluated on how well they deliver against the shifts. DMHIs that demonstrate clear alignment with these standards and frameworks are more likely to gain traction with commissioners, as they support strategic priorities, ensure compliance with national expectations, and contribute to the delivery of integrated, needs-led mental health care.

### 3.4 Timing

Timing was also considered to be a critical key in commissioning decisions, particularly when aligned with financial cycles such as year-end underspend. Commissioners often look for opportunities to allocate remaining budgets efficiently before the close of the financial year. This can make interventions with a clearly defined endpoint particularly attractive. Commissioners reflected that a common practice was to have an individual funding request approved for a set number of sessions, only for additional funding requests to follow. DMHI that contain a set number of modules/engagement may be reassuring to commissioning panels, eliminating the risk of financial creep and making it a compelling option for time-sensitive or end-of-year investment.

## 4 Discussion

The workshop highlighted the complexities, fragmented and variable nature of commissioning DMHI within the UK NHS. Funding for digital mental health, particularly for young people, is often limited. Implementation challenges such as workforce training, IT integration, and information governance further delay adoption, even for evidence-based tools. Without a standardised commissioning pathway for DMHIs, especially those targeting young people, innovators may struggle to identify who the decision-makers are and how best to position their intervention. Importantly, the workshop identified that there is no clear single route in, and that often building a local case of need/proof of efficacy within an individual service might be an easier first step to implementation. However, this lacks the consistency and future-proofing that would be gained by more dedicated national funding which would support wider adoption with more security of provision for future years.

Our workshop highlighted the critical role of engaging commissioners throughout the entire lifecycle of a DMHI. Early involvement during the prototype development phase helps ensure that the intervention is appropriately designed for integration with existing systems and IT infrastructure, while also accommodating the inevitable variability across NHS Trusts. It also facilitates the incorporation of key performance metrics aligned with service effectiveness, acceptability, and statutory targets set by NHS Trusts. During the research phase, commissioner engagement helps align outcome measures with real-world service evaluation priorities. Finally, involving commissioners in the early stages of commercialisation supports the development of targeted strategies that enhance the likelihood of successful implementation and adoption into routine practice. However, the current fragmented, localised “Trust by Trust” or “ICB by ICB” approach to evidence, governance, and procurement presents a significant challenge for companies. This highlights the need for a more collective approach to developing common standards, sharing evidence, and distributing risk across the system.

Organisations such as HINs can play an important enabling role to facilitate early conversations with commissioners, but they cannot fully mitigate the underlying system-level challenges that impede the adoption of digital innovation in mental health care. Additionally, the commissioners that attended our event all commented on how powerful our PPI lived experience voice was, noting this should be included in developing our business case and marketing materials, which highlights the importance of continued PPI, even beyond the research and development stage.

Ultimately, the successful implementation of DMHIs depends on a collaborative, cross-sector approach that brings together innovators, commissioners, service providers, and patient and public members. Strengthening these partnerships is essential to overcoming systemic barriers and ensuring that digital innovations can be effectively embedded into mental health care pathways.

## Data Availability

The original contributions presented in the study are included in the article/Supplementary Material, further inquiries can be directed to the corresponding author.

## References

[1] Saxena S Thornicroft G Knapp M Whiteford H. Resources for mental health: scarcity, inequity, and inefficiency. Lancet. (2007) 370(9590):878–89. 10.1016/S0140-6736(07)61239-2 17804062

[2] Schueller SM Torous J. Scaling evidence-based treatments through digital mental health. Am Psychol. (2020) 75(8):1093–104. 10.1037/amp0000654 33252947 PMC7709142

[3] Hollis C Falconer CJ Martin JL Whittington C Stockton S Glazebrook C Annual research review: digital health interventions for children and young people with mental health problems—a systematic and meta-review. J Child Psychol Psychiatry. (2017) 58(4):474–503. 10.1111/jcpp.12663 27943285

[4] Evidence standards framework for digital health technologies—NICE Guidance | [Internet]. (2018). Available online at: https://www.nice.org.uk/corporate/ecd7/chapter/how-to-meet-the-standards (Accessed September 9, 2025)

[5] Hollis C Loades M Hall CL. Digital technology- assessment and treatment. In: Thapar A Pine DS Cortese S Creswell C Ford TJ Leckman JF, editors. Rutter’s Child and Adolescent Psychiatry and Psychology. UK: John Wiley & Sons (2025). p. 633–51. ISBN: 978-1-119-90596-7

[6] Liu M Schueller SM. Moving evidence-based mental health interventions into practice: implementation of digital mental health interventions. Curr Treat Options Psychiatry. (2023) 10(4):1–13. 10.1007/s40501-023-00298-2

[7] Smith KA Blease C Faurholt-Jepsen M Firth J Van Daele T Moreno C Digital mental health: challenges and next steps. BMJ Ment Health. (2023) 26(1):e300670. 10.1136/bmjment-2023-300670 37197797 PMC10231442

[8] NHS Long Term Plan [Internet]. Available online at: https://www.longtermplan.nhs.uk/ (Accessed August 21, 2025)

[9] Hall CL Bergin ADG Rennick-Egglestone S. Research into digital health intervention for mental health: 25-year retrospective on the ethical and legal challenges. J Med Internet Res. (2024) 26(1):e58939. 10.2196/58939 39250796 PMC11420603

[10] Hall CL Davies EB Andren P Murphy T Bennett S Brown BJ Investigating a therapist-guided, parent-assisted remote digital behavioural intervention for tics in children and adolescents-’online remote behavioural intervention for tics’ (ORBIT) trial: protocol of an internal pilot study and single-blind randomised controlled trial. BMJ Open. (2019) 9(1):e027583. 10.1136/bmjopen-2018-027583 30610027 PMC6326281

[11] Hollis C Hall CL Khan K Le Novere M Marston L Jones R Online remote behavioural intervention for tics in 9-to 17-year-olds: the ORBIT RCT with embedded process and economic evaluation. Health Technol Assess Winch Engl. (2023) 27(18):1. 10.3310/CPMS3211 PMC10641713 37924247

[12] Hollis C Hall CL Jones R Marston L Novere ML Hunter R Therapist-supported online remote behavioural intervention for tics in children and adolescents in England (ORBIT): a multicentre, parallel group, single-blind, randomised controlled trial. Lancet Psychiatry. (2021) 8(10):871–82. 10.1016/S2215-0366(21)00235-2 34480868 PMC8460453

[13] Hollis C Hall CL Khan K Jones R Marston L Le Novere M Long term clinical and cost-effectiveness of a therapist-supported online remote behavioural intervention for tics (ORBIT) in children and adolescents: extended 12 and 18-month follow-up of a single-blind randomised controlled trial. J Child Psychol Psychiatry. (2023) 64(6):941–51. 10.1111/jcpp.13756 36649686

[14] Digital Therapy for Chronic tic Disorders and Tourette Syndrome: Early Value Assessment—Guidance—NICE. NICE (2025). Available online at: https://www.nice.org.uk/guidance/hte25 (Accessed September 9, 2025).

[15] Gov.uk [Internet]. “Innovator passports” set to accelerate cutting-edge NHS care. Available online at: https://www.gov.uk/government/news/innovator-passports-set-to-accelerate-cutting-edge-nhs-care (Accessed August 21, 2025)

[16] Brantnell A Temiz S Baraldi E Woodford J von Essen L. Barriers to and facilitators of the implementation of digital mental health interventions as perceived by primary care decision makers: content analysis of structured open-ended survey data. JMIR Hum Factors. 2023 10(1):e44688. 10.2196/44688 37358902 PMC10337378

[17] www.england.nhs.uk/south/wp-content/uploads/sites/6/2017/03/faqs-mental-health-financial-planning.pdf.

[18] i-THRIVE—Implementing the THRIVE Framework [Internet]. Available online at: https://implementingthrive.org/ (Accessed July 8, 2025)

